# Incorporating capture heterogeneity in the estimation of autoregressive coefficients of animal population dynamics using capture–recapture data

**DOI:** 10.1002/ece3.6642

**Published:** 2020-08-31

**Authors:** Pedro G. Nicolau, Sigrunn H. Sørbye, Nigel G. Yoccoz

**Affiliations:** ^1^ Department of Mathematics and Statistics Faculty of Science and Technology UiT The Arctic University of Norway Tromso Norway; ^2^ Department of Arctic and Marine Biology Faculty of Biosciences, Fisheries and Economics UiT The Arctic University of Norway Tromso Norway

**Keywords:** abundance, capture probability, closed population models, density dependence, INLA, process variance

## Abstract

Population dynamic models combine density dependence and environmental effects. Ignoring sampling uncertainty might lead to biased estimation of the strength of density dependence. This is typically addressed using state‐space model approaches, which integrate sampling error and population process estimates. Such models seldom include an explicit link between the sampling procedures and the true abundance, which is common in capture–recapture settings. However, many of the models proposed to estimate abundance in the presence of capture heterogeneity lead to incomplete likelihood functions and cannot be straightforwardly included in state‐space models. We assessed the importance of estimating sampling error explicitly by taking an intermediate approach between ignoring uncertainty in abundance estimates and fully specified state‐space models for density‐dependence estimation based on autoregressive processes. First, we estimated individual capture probabilities based on a heterogeneity model for a closed population, using a conditional multinomial likelihood, followed by a Horvitz–Thompson estimate for abundance. Second, we estimated coefficients of autoregressive models for the log abundance. Inference was performed using the methodology of integrated nested Laplace approximation (INLA). We performed an extensive simulation study to compare our approach with estimates disregarding capture history information, and using R‐package VGAM, for different parameter specifications. The methods were then applied to a real data set of gray‐sided voles *Myodes rufocanus* from Northern Norway. We found that density‐dependence estimation was improved when explicitly modeling sampling error in scenarios with low process variances, in which differences in coverage reached up to 8% in estimating the coefficients of the autoregressive processes. In this case, the bias also increased assuming a Poisson distribution in the observational model. For high process variances, the differences between methods were small and it appeared less important to model heterogeneity.

## Graphical abstract

We assessed the importance of including capture history information in the estimation of density dependence from capture–recapture data. We performed an extensive simulation study, evaluating the performance of different methods in the estimation of different population processes. We found that disregarding sampling error can bias the density‐dependence estimates in low variance autoregressive population models.

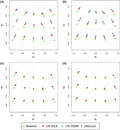

## 1 INTRODUCTION

Models used to analyze population dynamics include a combination of density dependence and environmental effects. Ignoring the uncertainty in abundance estimates biases estimates of the strength of density dependence, and different approaches exist to achieve better accuracy (see Lebreton & Gimenez, 2012 for a review). In particular, state‐space models combining an observation model—linking the observations such as counts to the true abundance—and a process model—describing the processes driving population dynamics—have become a standard approach in many analyses (Dennis & Taper, 1994). However, these models rarely include an explicit model of the link between how counts were obtained and true abundance, often relying on a nonspecific observation model such as log normal or Poisson distribution (for instance, Ono, Langangen, & Chr. Stenseth, 2019, but see below).

Capture–recapture methods have been extensively used to estimate abundance and density dependence, and many methods have been developed to incorporate different sources of variability into capture probability estimation, such as environmental information, survival, or trophic interactions (Barker, Fletcher, & Scofield, 2002; Lebreton & Gimenez, 2012; Schofield & Barker, 2008; Yackulic, Korman, Yard, & Dzul, 2018). Estimating abundance is a challenging statistical problem (Link, 2003), and heterogeneity in capture probabilities can lead to large biases in abundance estimates when using models assuming no heterogeneity (Carothers, 1973; Otis, Burnham, White, & Anderson, 1978). However, many of the models that have been proposed to estimate abundance in the presence of heterogeneity do not lead to observation models that can be included in state‐space models as they do not lead to likelihood functions in a closed form (Chao & Huggins, 2006; Huggins & Hwang, 2011).

Many studies investigating density dependence have used simple process models such as the Gompertz model—that is, a model which is a first‐order autoregressive model on a log scale (Ono et al., 2019; Thibaut & Connolly, 2019). However, ecological processes such as trophic interactions (Bjørnstad, Falck, & Stenseth, 1995) or intrinsic ecological properties such as age structure (Lande, Engen, & Sæther, 2002) may lead to more complex process models such as a second‐order autoregressive model (AR(2)). An important case is the population cycles observed in many small mammal populations, particularly in northern environments (Bjørnstad & Chr. Stenseth, 1999; Elton, 1924; Stenseth, 1999). These quasi‐periodic fluctuations are quite well approximated by AR(2) models on a logarithmic scale (Bjørnstad et al., 1995). Whereas most analyses have ignored the uncertainty in abundance estimates (Bjørnstad et al., 1995), some have used state‐space models (Cornulier et al., 2013; Ims, Yoccoz, & Killengreen, 2011; Kleiven, Henden, Ims, & Yoccoz, 2018; Stenseth et al., 2003). However, approaches using a capture–recapture framework and including capture heterogeneity have relied on integrating out random effects describing capture heterogeneity (King, Brooks, & Coulson, 2008; Schofield & Barker, 2013) and using superpopulation data augmentation (Royle, 2008); these approaches did not consider the conditional likelihood approach to estimating population size, which can easily handle, for example, individual covariates (Huggins & Hwang, 2011). Moreover, a fully MCMC‐based Bayesian approach is computationally intensive on large data sets and requires that careful considerations are given to choices of priors and superpopulation sizes for data augmentation (Royle, Dorazio, & Link, 2007).

Here, we investigated the performance of an intermediate approach between ignoring uncertainty in abundance estimates (i.e., using the raw population counts) and fully specified state‐space models. Specifically, we first used a multinomial observation model to estimate capture probabilities followed by estimating abundance at each time point using the Horvitz–Thompson estimator (Horvitz & Thompson, 1952). Second, we fitted an AR(2) process model to the log abundance to estimate direct and delayed density dependence given by the first and second coefficients of the AR(2) model, respectively. Both estimation steps were performed in a unified way, incorporating the models within the general class of latent Gaussian models (Rue, Martino, & Chopin, 2009). Full Bayesian inference was then obtained using the methodology of integrated nested Laplace approximation (INLA) (Rue et al., 2009, 2017).

We based our analyses on a large‐scale study of population dynamics of the dominant small mammal species in northern Fennoscandia, the gray‐sided vole *Myodes rufocanus* (Ims et al., 2011). This species shows large fluctuations with a 4‐ to 5‐year periodicity (Ims et al., 2011; Marolla et al., 2019). We monitored populations of gray‐sided voles along a 155‐km gradient from coast to inland, using live capture–recapture methods, starting in 2000. Previous analyses have shown that there was large heterogeneity in capture probabilities (Yoccoz & Ims, 2004). In this rodent study, the goal was to understand spatial patterns of population dynamics, assessing potential seasonal effects on the density‐dependence estimates. For this, we first needed to assess the robustness of using an approach based on estimated abundances but without implementing a full state‐space model. In this paper, we therefore use a simulation study built around the case study (adaptable to other situations from the code provided) to assess the estimation accuracy of the density dependence, both including and excluding capture history information.

The structure of this paper is as follows. Section 2 provides our methodological background to analyze capture–recapture data and describes the Bayesian framework to perform parameter estimation. This includes using INLA to estimate individual capture probabilities and the direct and delayed density dependence given by the coefficients of AR(2) models. Section 3 contains an extensive simulation study, investigating how density‐dependence estimates are influenced when individual capture probabilities are taken into account. In Section 4, we study the population cycles of gray‐sided voles. We first compare different observation models in estimating individual capture probabilities and then assess whether incorporation of individual capture probabilities influences density‐dependence estimates. A summary and concluding remarks are given in Section 5.

## 2 METHODOLOGY

Capture–recapture experiments are important to assess heterogeneity in individual capture probabilities. This section describes our approach to incorporate capture–recapture information in the estimation of density dependence. First, we define an observation model in which capture probabilities are modeled in terms of individual features and then used to estimate abundance. Second, we fit an AR(2) process model to the estimated log abundance to assess density dependence. When using state‐space approaches, the parameters of the observation and process model are estimated simultaneously. This is not possible in our case as the capture probabilities are estimated based on a conditional multinomial likelihood, due to individuals that were not observed. Instead, we apply a sequential approach, first estimating the capture probabilities and then the AR(2) coefficients. This allows us to use an explicit sampling model to estimate capture probabilities, instead of assuming that the observed counts have a Poisson or log normal distribution. The given sequential approach is computationally efficient using the R‐INLA package which is freely available at www.r‐inla.org.

### 2.1 Statistical background on capture–recapture data

Assume a closed population with a total of *N* individuals and a capture–recapture experiment with *τ* capture sessions. Let

$$w_{i}^{\prime }=\left(w_{i1},\ldots ,w_{i\tau }\right),\;i=1,\ldots ,N,$$

denote the capture history for the *i*th individual. If *w_ij_* = 1, the individual was captured at the *j*th capture session, while *w_ij_* = 0 otherwise, that is, *w_ij_* ~ Bernoulli(*p_ij_*), *j* = 1, …, *τ*. For each individual, the probability of a given capture history is then

$$p_{w_{i}}=\prod_{j=1}^{\tau }p_{\mathrm{ij}}^{w_{\mathrm{ij}}}{\left(1‐p_{\mathrm{ij}}\right)}^{1‐w_{\mathrm{ij}}},\;i=1,\ldots ,N.$$

Assuming that all individuals are captured independently, the complete likelihood becomes

$$L\left(N,\left\{p_{\mathrm{ij}}\right\}|\left\{w_{\mathrm{ij}}\right\}\right)=\prod_{i=1}^{N}\prod_{j=1}^{\tau }p_{\mathrm{ij}}^{w_{\mathrm{ij}}}{\left(1‐p_{\mathrm{ij}}\right)}^{1‐w_{\mathrm{ij}}},$$

where both *N* and the set of probabilities {*p_ij_*} are unknown. Due to the unknown number of noncaptured individuals, computation of the likelihood is unfeasible. This is a well‐known problem (Huggins & Hwang, 2011) and requires alternative strategies to perform parameter estimation.

A commonly applied approach is to maximize the conditional likelihood for the *n* individuals that were captured at least once. Let *c_ik_*, *k* = 0, …, 2*^τ^* − 1, denotes the probability that the capture history of individual *i* is equal to category *k*. The different categories are defined by all possible permutations of the capture session vector, giving a total of *m* = 2*^τ^* − 1 categories for the captured individuals.

From here onwards, we will refer to data sets with only two capture events, in which mortality and emigration are disregarded considering capture events on adjacent days. The event that an individual is never captured is then defined as category 0, while the categories 1, 2, and 3 refer to the capture histories (1,0), (0,1), and (1,1), respectively. To perform parameter estimation, we need to make realistic assumptions on the capture probabilities for different capture sessions. Otis et al. (1978) propose a total of eight different models characterizing capture probabilities for different sessions depending on time, behavior, and homogeneity of the individuals, also including combinations of these three factors. Here, we consider a heterogeneity model including a temporal effect, *M*th. This implies that the capture probabilities depend on different features of the individuals. Further, we assume that the capture probability on the first and second capture sessions is independent. The probabilities for the different categories are then specified as

(1) $$c_{i0}=\left(1‐p_{i1}\right)\left(1‐p_{i2}\right),\;c_{i1}=p_{i1}\left(1‐p_{i2}\right),\;c_{i2}=\left(1‐p_{i1}\right)p_{i2},\;c_{i3}=p_{i1}p_{i2},\;i=1,\ldots ,N.$$

To estimate abundance based on individuals that were captured, we use the Horvitz–Thompson estimator (Horvitz & Thompson, 1952)

(2) $$\hat{N}=\sum_{i=1}^{n}{\left(1‐{\hat{c}}_{i0}\right)}^{‐1},$$

where ${\hat{c}}_{i0}$ denotes the estimated probability that individual *i* was not captured. This probability is estimated using a regression model as explained in the next section.

### 2.2 A multinomial capture–recapture regression model including a Poisson transformation

An important question in analyzing population processes from capture–recapture data is whether features of the captured individuals give valuable information in further analysis of density dependence. To estimate the probabilities in (2), it is natural to assume a multinomial regression model for the captured individuals, incorporating covariate information which helps to separate different capture categories. We define the vector $Y_{i}^{\prime }$ = (*Y_i_*_1_, …, *Y_im_*), where *Y_ik_* = 1 for an individual classified to category *k*, while the remaining elements of **Y** *_i_* are 0. Each of the vectors **Y**_1_, …, **Y** *_n_* has a multinomial distribution. Based on (1), probabilities for the *m* = 3 observed categories are defined by ${\tilde{c}}_{\mathrm{ik}}=c_{\mathrm{ik}}/\left(1‐c_{i0}\right),\;k=1,\ldots ,m$, ensuring that the probabilities sum to 1. These probabilities can then be modeled in terms of observed individual features such as weight, sex, and age.

We denote the individual features or covariates by $z_{r}^{\prime }=\left(z_{1r},\ldots ,z_{\mathrm{nr}}\right)$. Further, we define the linear predictor

(3) $$V_{\mathrm{ik}}=\sum_{r=1}^{v}\gamma_{\mathrm{kr}}z_{\mathrm{ir}},\;i=1,\ldots ,n,\;k=1,\ldots ,m,$$

where the coefficient *γ_kr_* is specific for category *k* and covariate *r*, while *v* is the number of covariates. The scaled probabilities for the captured individuals are then expressed as

(4) $${\tilde{c}}_{\mathrm{ik}}=\frac{e^{V_{\mathrm{ik}}}}{\sum_{k=1}^{m}e^{V_{\mathrm{ik}}}},\;i=1,\ldots ,n,\;k=1,\ldots ,m.$$

The resulting multinomial likelihood is

(5) $$L_{M}\left(\gamma_{1},\ldots ,\gamma_{v}|y_{1},\ldots ,y_{n}\right)\propto \prod_{i=1}^{n}\prod_{k=1}^{m}{\left({\tilde{c}}_{\mathrm{ik}}\right)}^{y_{\mathrm{ik}}},$$

where $\gamma_{i}^{\prime }=\left(\gamma_{1r},\;\ldots ,\;\gamma_{\mathrm{mr}}\right)$, *r* = 1, …, *v*. Notice that in maximizing (5), the denominator of ${\tilde{c}}_{\mathrm{ik}}$ does not simplify using the ordinary logarithmic transformation. It is therefore common to apply the well‐known multinomial Poisson transformation (Baker, 1994) in which the likelihood is rewritten as

$$L_{P}\left(\gamma_{1},\ldots ,\gamma_{v},{\beta |y}_{1},\ldots ,y_{n}\right)\propto \prod_{i=1}^{n}\prod_{k=1}^{m}e^{‐\mu_{\mathrm{ik}}}\mu_{\mathrm{ik}}^{y_{\mathrm{ik}}}.$$

Here, $\mu_{\mathrm{ik}}=e^{V_{\mathrm{ik}}+\beta_{i}}$ represents the rate of a Poisson distributed random variable *Y_ik_*. The given transformation from a multinomial likelihood to the Poisson likelihood introduces auxiliary parameters **β**′ = (*β*_1_, …, *β_n_*), in which *β_i_* is proportional to $\ln\left(\sum_{k=1}^{m}e^{V_{\mathrm{ik}}}\right)$. This is just a technical detail to make the approximation work correctly. The likelihood *L_P_*(.) is proportional to *L_M_*(.) and gives the same maximum‐likelihood estimates for the coefficient vectors **γ** *_r_*. The resulting regression model is then summarized in terms of linking the expectation of the Poisson variables to the linear predictor using the log transform, that is,

(6) $$\ln\left(E\left(Y_{\mathrm{ik}}\right)\right)=\ln\left(\mu_{\mathrm{ik}}\right)=\sum_{r=1}^{v}\gamma_{\mathrm{kr}}z_{\mathrm{ir}}+\beta_{i}+\epsilon_{i},\;i=1,\ldots ,n,\;k=1,\ldots ,m,$$

where $\epsilon_{i}∼N\left(0,\kappa^{‐1}\right)$ denotes small independent random error terms.

In fitting the given model to a data set, the vectors ${\left\{\gamma_{r}\right\}}_{r=1}^{v}$ will not be identifiable. However, in our case we only need estimates of the differences in these coefficients as these represent ratios of log probabilities between the different categories. For categories *k* and *l*, we notice that

$$\ln\left(\frac{{\tilde{c}}_{\mathrm{ik}}}{{\tilde{c}}_{\mathrm{il}}}\right)=V_{\mathrm{ik}}‐V_{\mathrm{il}}=\sum_{r=1}^{v}\left(\gamma_{\mathrm{kr}}‐\gamma_{\mathrm{lr}}\right)z_{\mathrm{ir}}.$$

In estimating the parameters of the model, this implies that the auxiliary parameters and error terms disappear, but these are still included in fitting (6) to a data set. In the case of assuming (1), the estimated individual probabilities are then given by

(7) $$\ln\left(\frac{{\hat{p}}_{i1}}{1‐{\hat{p}}_{i1}}\right)=\sum_{r=1}^{v}\left({\hat{\gamma }}_{3r}‐{\hat{\gamma }}_{2r}\right)z_{\mathrm{ir}},$$

(8) $$\ln\left(\frac{{\hat{p}}_{i2}}{1‐{\hat{p}}_{i2}}\right)=\sum_{r=1}^{v}\left({\hat{\gamma }}_{3r}‐{\hat{\gamma }}_{1r}\right)z_{\mathrm{ir}},$$

or equivalently

(9) $${\hat{p}}_{i1}=\frac{e^{\sum_{r=1}^{v}\left({\hat{\gamma }}_{3r}‐{\hat{\gamma }}_{2r}\right)z_{\mathrm{ir}}}}{1+e^{\sum_{r=1}^{v}\left({\hat{\gamma }}_{3r}‐{\hat{\gamma }}_{2r}\right)z_{\mathrm{ir}}}},$$

(10) $${\hat{p}}_{i2}=\frac{e^{\sum_{r=1}^{v}\left({\hat{\gamma }}_{3r}‐{\hat{\gamma }}_{1r}\right)z_{\mathrm{ir}}}}{1+e^{\sum_{r=1}^{v}\left({\hat{\gamma }}_{3r}‐{\hat{\gamma }}_{1r}\right)z_{\mathrm{ir}}}}.$$

These probabilities are then used to estimate ${\hat{c}}_{i0}$ in (2).

### 2.3 Implementation using a Bayesian framework

To fit (6) to a data set and estimate the capture probabilities, we choose to apply a Bayesian approach. This implies that all parameters in (6) are viewed as random variables. Specifically, the resulting regression model can be incorporated within the computational framework of latent Gaussian models. This is a flexible class of three‐stage hierarchical models, which can be analyzed in a unified way using INLA. Subsequently, the model in (6) is reformulated in terms of having conditionally independent observations, given a latent field and hyperparameters.

The three stages of a latent Gaussian model are expressed as follows, where *π*(.) is generic notation for probability densities:

1. The first stage specifies the likelihood where the observations are assumed conditionally independent given a latent field **x** and hyperparameters **θ**. In our case, let $y^{\prime }=\left(y_{1}^{\prime },\ldots ,y_{n}^{\prime }\right)$ denote the stacked vector of the *m* categories for the *n* individuals. The likelihood is then expressed as

$$L\left(x,\theta |y\right)=\prod_{i=1}^{\mathrm{nm}}\pi \left(y_{i}|x_{i},\theta \right).$$

- The latent field **x** collects all random variables of the linear predictor

(11) $$\begin{array}{c} x=\left\{\gamma_{1},\ldots ,\gamma_{v},\beta ,\epsilon \right\} \end{array},$$

where we could also include the predictor itself. The latent field models the dependency structure of the observations and is assigned a multivariate Gaussian prior

$$\pi \left(x|\theta \right)∼N\left(0,Q^{‐1}\left(\theta \right)\right).$$

The precision (inverse covariance) matrix **Q** is typically sparse such that **x** has Markov properties and is then referred to as a Gaussian Markov random field.

- The hyperparameters **θ** of a latent Gaussian model are usually assigned non‐Gaussian priors. Here, we only have one hyperparameter being the precision parameter of the random error terms, *θ* = *κ*. This parameter is assigned a penalized complexity prior (Simpson, Rue, Riebler, Martins, & Sørbye, 2017), implying that *κ* ^−1/2^ has an exponential density.

The joint posterior for all elements of the latent field and the additional hyperparameter is then described as

$$\pi \left(x,\theta |y\right)\propto \prod_{i=1}^{\mathrm{nm}}\pi \left(y_{i}|x_{i},\theta \right)\pi \left(x|\theta \right)\pi \left(\theta \right).$$

The main interest is to calculate the marginal posteriors for each of the latent field components and each of the hyperparameters.

For the multinomial model, INLA is used to estimate the marginal posteriors for all the coefficients

$$\pi (\gamma_{\mathrm{kr}}|y),\;k=1,\ldots ,m,\;r=1,\ldots ,v.$$

These provide posterior mean estimates of the differences *γ_kr_* – *γ_lr_*, which are used to estimate the individual capture probabilities and the abundance by (2).

### 2.4 Estimating density dependence

Our final step is to fit a process model to study population dynamics of a species. Specifically, we focus on estimating density dependence by fitting an AR(2) model to a given time series, reflecting the population cycle for the relevant species. Let ln(*N_t_*) denote the true log abundance at time *t*. The AR(2) model is then defined by

(12) $$\ln\left(N_{t}\right)=\ln\left(\eta \right)+\phi_{1}\ln\left(N_{t‐1}\right)+\phi_{2}\ln\left(N_{t‐2}\right)+\epsilon_{t},\;t=1,\ldots ,T,$$

where ln(*η*) denotes an offset, while the noise terms are independent Gaussian variables, $\epsilon_{t}∼N\left(0,\sigma_{\epsilon }^{2}\right)$. *T* denotes the length of the time series, while the coefficients *ϕ*_1_ and *ϕ*_2_ characterize the direct and delayed density dependence of the series. The given process is stationary when $‐1\leq \phi_{2}\leq 1‐\left|\phi_{1}\right|<1$ and has pseudoperiodic behavior when $\phi_{1}^{2}+4\phi_{2}\leq 0$. Estimation of the coefficients of AR(2) is not influenced by the offset ln(*η*). This implies that if the number of captured individuals at different time points is proportional to the underlying true abundance, we would get identical parameter estimates.

The AR(2) model is fitted within the framework of latent Gaussian models using INLA. In this case, the model has three hyperparameters, including $\kappa =\sigma_{\epsilon }^{‐2}$ and the coefficients *ϕ*_1_ and *ϕ*_2_. These parameters are all assigned PC priors (Simpson et al., 2017; Sørbye & Rue, 2017). Of main interest is to study how the estimates of *ϕ*_1_ and *ϕ*_2_ vary when capture heterogeneity is accounted for using the multinomial observational model.

Often, simplifying assumptions regarding the data generating process are made, for example, by assuming a Poisson process (Stenseth et al., 2003) or a log‐normal distribution (Santin‐Janin et al., 2014) for the observed counts. These assumptions can be implicit while defining the observation models in state‐space approaches. We study the Poisson distribution assumption in an additional step also fitted using INLA. The log rate of the assumed underlying Poisson process for the abundance is expressed in terms of the linear predictor

(13) $$\lambda_{t}=\ln\left(E\left(N_{t}\right)\right)=\beta_{0}+e_{t},\;t=1,\ldots ,T.$$

Here, *β*_0_ denotes an intercept, while *e*_1_, …, *e_T_* denotes independent and identically distributed random variables, $e_{i}∼N\left(0,\kappa_{e}^{‐1}\right)$. These error terms are included to model random variation as a function of time. As detailed in the next section, the AR(2) model will be fitted either to the estimated log abundance $\ln\left({\hat{N}}_{1}\right),\ldots ,\ln\left({\hat{N}}_{T}\right)$ or to the posterior means of the log rates of the corresponding Poisson process, denoted ${\hat{\lambda }}_{1},\ldots ,{\hat{\lambda }}_{T}$.

## 3 SIMULATION STUDY COMPARING METHODS TO ESTIMATE DENSITY DEPENDENCE

This section provides an extensive simulation study to assess how the inclusion of capture history information influences estimation of density dependence. We start by simulating data to approximate a realistic capture–recapture sampling scenario. The underlying log population of the sampled species is generated as an AR(2) process in time, using different fixed combinations of the coefficients (*ϕ*_1_, *ϕ*_2_) and the innovation variance $\sigma_{\epsilon }^{2}$, from here onwards referred to as the (population) process variance. Each resulting individual is then assigned a random weight, and a two‐day capture history according to a multinomial model with probabilities defined by (1). We then fit an AR(2) process model to the estimates of log abundance or log rates obtained by different methods. These different methods are described in Section 3.1, while Section 3.2 specifies the simulation procedure and the method performance criteria used. Finally, Section 3.3 provides simulation results and an evaluation of the different methods.

### 3.1 Estimation methods

**FIGURE 1:**
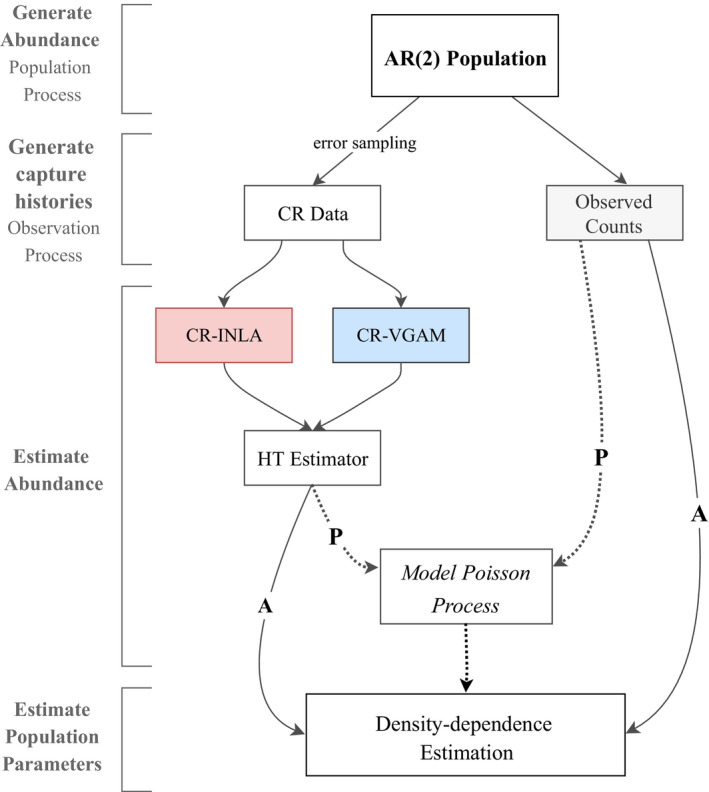
Methodological flowchart

An overview of the different estimation methods used in the simulation study is given in Figure 1. The left‐hand side of the figure shows the additional steps needed to implement the observation model, incorporating sampling error in terms of capture history information. We employ two methods of estimating individual capture probabilities. The first is described in Sections 2.2 using INLA (method: CR‐INLA) and corresponds to our suggested approach. The second, for comparison, estimates individual capture probabilities using the R‐package VGAM (Yee, 2019). Among other utilities, the VGAM (vector generalized additive model) framework can be used to analyze closed population capture–recapture data, allowing the incorporation of individual covariates while using the conditional likelihood (Yee, Stoklosa, & Huggins, 2015). This application of VGAM allows for a flexible and efficient estimation of capture probabilities for all of the eight heterogeneity models given by Otis et al. (1978) (method: CR‐VGAM). From the estimated capture probabilities from either of the two methods, we proceed to estimate the true log abundance using the Horvitz–Thompson estimator in (2). At this point, we have two possible variants in estimating density dependence: We either fit the AR(2) model to the times series of estimated log abundance ${\left\{\ln\left({\hat{N}}_{t}\right)\right\}}_{t=1}^{T}$ (A variant), or we fit the AR(2) model to the corresponding estimated log rate of a Poisson process, ${\left\{{\hat{\lambda }}_{t}\right\}}_{t=1}^{T}$ (P variant). The right‐hand side of Figure 1 illustrates the approach disregarding capture history, fitting the AR(2) model directly to the observed log counts, or to the log rate of the corresponding Poisson process (method: ObsCount). Finally, the performance of the different estimation methods is compared with the results fitting the AR(2) model to the true generated log abundance or estimated log rate (method: Baseline).

### 3.2 Simulation procedure

For each combination of AR(2) coefficients, (*ϕ*_1_, *ϕ*_2_), we generated *M* = 200 time series. Specifically, we chose *ϕ*_1_ ∈ (−1, −0.5, 0, 0.5, 1) and *ϕ*_2_ ∈ (−0.8, −0.5, −0.2), giving a total of fifteen combinations of the coefficients. These combinations ensure that the resulting generated time series were stationary, also having pseudoperiodic behavior. To investigate the effect of varying the process variance of the AR(2) process, we further compared density‐dependence estimates for the values $\sigma_{\epsilon }^{2}\in \left(0.04,0.08,0.16,0.32,0.64\right)$. The details of the simulation procedure can be described in the following steps:

1. Generate the series ${\left\{\ln\left(N_{t}\right)\right\}}_{t=1}^{T}$ according to (12) where *T* = 20, using different fixed combinations of (*ϕ*_1_, *ϕ*_2_). To remove the effect of sample size on the estimation of capture probability, we assumed that *E*(*N_t_*) = 20 by using an offset $\ln\left(\eta \right)=\ln\left(20\right)‐\frac{1}{2}\text{Var}\left(\ln\left(N_{t}\right)\right)$. The series was rounded to give integer values for ${\left\{N_{t}\right\}}_{t=1}^{T}$, representing the abundance of an animal population. The total number of individuals generated for one simulated AR(2) process was then $\tilde{N}=\sum_{t=1}^{T}N_{t}$.
2. For each of the $\tilde{N}$ individuals, we generated a random weight

$$z_{\mathrm{it}}|\mu_{t}∼\text{Log}\;\text{normal}\left(\ln\left(\mu_{t}\right),\ln\left(\sigma_{w}\right)\right),$$

where *σ_w_* = 1.2, while *μ_t_* ~ Log normal(ln(30), ln(5)). The weight was then scaled by the sample standard deviation of the generated weights to make it dimensionless. The resulting variable was used as an individual‐specific covariate in (3). In this context, weight is a proxy for detectability. We varied the expected value of weight with time to model varying detectability, reflecting changes in the composition of the population at different time points. Thus, the varying mean reflects biological variation which we considered more realistic than assuming constant capture probabilities for different time points. The parameters relating to the weight distribution were here chosen to illustrate this biological variation.

- Assume a temporal effect *M*th for the capture–recapture process with *τ* = 2. To assign a capture history to each individual, we first assumed that the capture probabilities for days 1 and 2 were *p_i_*_1_ ≡ *p*_1_ = 0.55 and *p_i_*_2_ ≡ *p*_2_ = 0.75 for the total generated population. These probabilities were used to find reasonable values for the specific coefficients for the observed categories in terms of

$$\gamma_{31}‐\gamma_{21}=\ln\left(\frac{p_{1}}{1‐p_{1}}\right)\;\text{and}\;\gamma_{31}‐\gamma_{11}=\ln\left(\frac{p_{2}}{1‐p_{2}}\right).$$

The final individual capture probabilities were then computed according to (9)–(10) including the generated random weight as a covariate, implying v=1.

- Remove individuals with capture history according to category 0 (undetected).
- Estimate abundance using each of the methods described in Section 3.1 and fit an AR(2) model to the resulting time series including both the A and P variants.

The choices made in this simulation study intended to approximate the characteristics of a real ecological data set. Specifically, we have chosen to simulate rather short time series, having similar length as the real data set used in Section 4. Also, the initial capture probabilities for day 1 and day 2 were close to the proportions of captured individuals in the real data set (being 0.55 and 0.77, respectively).

Our next step was to apply INLA and fit the AR(2) process model to the generated time series. This provided estimates of the marginal posterior distributions for the two AR coefficients *ϕ*_1_ and *ϕ*_2_, for all approaches. Based on the posterior distributions, we could then calculate summary statistics, including the posterior mean of the coefficients, the standard deviations, and credible intervals. To evaluate and compare the quality of the different density‐dependence estimates, we computed the estimated root mean‐squared error (RMSE), defined by

$$\text{RMSE}\left({\hat{\phi }}_{k}\right)=\sqrt{\frac{1}{M}\sum_{i=1}^{M}{\left({\hat{\phi }}_{k}‐\phi_{k}\right)}^{2}},\;k=1,2.$$

Here, ${\hat{\phi }}_{k}$ denotes the posterior mean estimate of the *k*th AR coefficient and $\phi_{k}$ denotes the true value of that coefficient, while *M* is the number of simulations. We also compared the frequentist coverage properties using the different approaches. This corresponded to finding the proportion of times the true AR coefficient was inside the *M* estimated 95% credible intervals. This means we would expect a coverage of 0.95 for an unbiased AR coefficient estimator.

### 3.3 Simulation results

Table 1 displays the average performance in terms of coverage and RMSE for the different methods used to estimate density dependence, including the two variants A and P. The averages were computed across all the given combinations of (*ϕ*_1_, *ϕ*_2_) and for each of the five fixed values of $\sigma_{\epsilon }^{2}$. Due to the short time‐series length, coverage using the Baseline method will not achieve the nominal level of 0.95 (only nominal for the A variant). It is well known that estimators for the coefficients of AR processes are biased for small sample sizes (Shaman & Stine, 1988). Furthermore, the Baseline method for the P variant is not optimal considering it is based on the true log counts rather than alternatively generating true log rates of a Poisson process.

**TABLE 1:** The estimated average coverage and RMSE for all combinations of (*ϕ*_1_, *ϕ*_2_) in the four methods, using five levels of $\sigma_{\epsilon }^{2}$. The AR(2) process was either fitted to the log abundance (*A*) or the log rate of the corresponding Poisson process (*P*)

		Coverage				RMSE			
		*ϕ_1_*		*ϕ_2_*		*ϕ_1_*		*ϕ_2_*	
Method	$\sigma_{\epsilon }^{2}$	*A*	*P*	*A*	*P*	*A*	*P*	*A*	*P*
Baseline	0.04	0.91	0.85	0.88	0.86	0.21	0.40	0.20	0.36
CR‐INLA	0.04	0.83	0.87	0.80	0.85	0.27	0.38	0.27	0.35
CR‐VGAM	0.04	0.80	0.83	0.77	0.83	0.29	0.40	0.28	0.37
ObsCount	0.04	0.77	0.81	0.75	0.82	0.31	0.42	0.29	0.38
Baseline	0.08	0.91	0.92	0.89	0.90	0.20	0.26	0.20	0.25
CR‐INLA	0.08	0.87	0.89	0.85	0.87	0.25	0.27	0.24	0.26
CR‐VGAM	0.08	0.86	0.88	0.84	0.86	0.26	0.29	0.25	0.27
ObsCount	0.08	0.84	0.87	0.82	0.85	0.27	0.31	0.26	0.29
Baseline	0.16	0.92	0.91	0.88	0.88	0.20	0.22	0.20	0.21
CR‐INLA	0.16	0.89	0.89	0.86	0.86	0.23	0.24	0.22	0.23
CR‐VGAM	0.16	0.88	0.88	0.86	0.85	0.24	0.25	0.23	0.24
ObsCount	0.16	0.87	0.88	0.85	0.85	0.24	0.25	0.23	0.24
Baseline	0.32	0.91	0.91	0.88	0.88	0.21	0.21	0.20	0.21
CR‐INLA	0.32	0.89	0.89	0.86	0.87	0.23	0.23	0.22	0.22
CR‐VGAM	0.32	0.88	0.88	0.87	0.86	0.23	0.23	0.22	0.23
ObsCount	0.32	0.88	0.88	0.86	0.86	0.23	0.24	0.23	0.23
Baseline	0.64	0.91	0.90	0.89	0.87	0.21	0.22	0.20	0.21
CR‐INLA	0.64	0.88	0.88	0.85	0.85	0.23	0.23	0.22	0.22
CR‐VGAM	0.64	0.88	0.87	0.84	0.84	0.23	0.23	0.23	0.23
ObsCount	0.64	0.87	0.87	0.84	0.84	0.23	0.24	0.23	0.23

The differences for the different methods were rather small, except for the two lowest process variance levels where there was a clear benefit from including capture history. CR‐INLA provided the highest coverage, followed by CR‐VGAM and ObsCount. Using CR‐INLA, the coverage was within the range (0.83–0.89) for *ϕ*_1_ and within the range (0.80–0.86) for *ϕ*_2_. Further, the results indicated that fitting the AR(2) model to the log rate of a Poisson process (P variant) provided generally higher coverage than using the A variants. When comparing the different methods using RMSE, which considers both bias and variance, we see that CR‐INLA had the smallest error in all cases, while the method ObsCount had the largest error. Again, the differences between the methods were very small except for the lowest levels of the process variance. In general, RMSE was reduced for all methods as the process variance increased. Moreover, RMSE was higher for the P variants compared with the A variants at the two lowest process variance levels, using all methods. This was due to both an increased variance and bias, which explains why the P variants had higher coverage.

**FIGURE 2:**
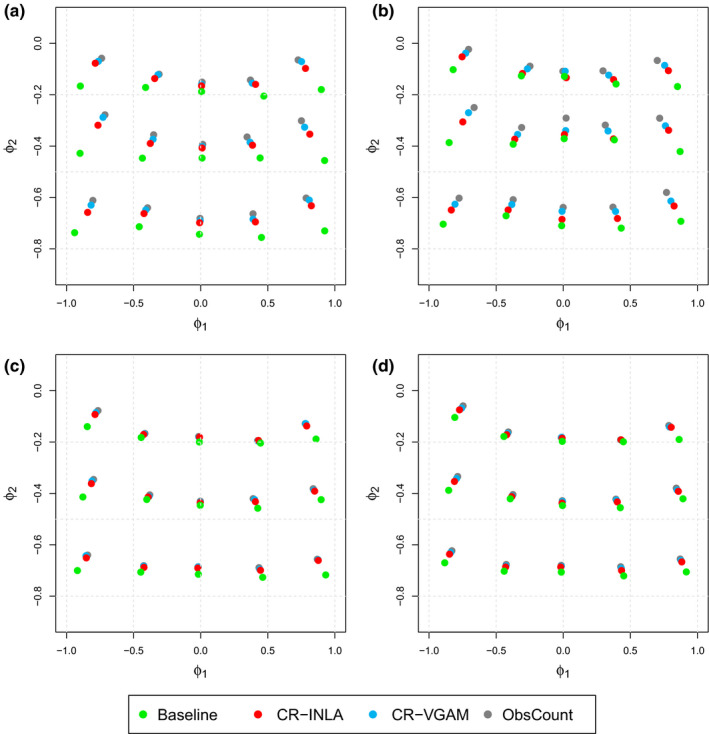
Posterior mean estimates of *ϕ*_1_ and *ϕ*_2_, for the A variants on the left (panels a and c) and P variants on the right (panels b and d). The points of intersection of the dotted gray lines correspond to the true parameter values. The intersections, at which each set of dots lean to, correspond to the true value of that given set. Panels a and b show results when $\sigma_{\epsilon }^{2}=0.08$, whereas c and d correspond to $\sigma_{\epsilon }^{2}=0.32$

The estimation bias of the different methods can be assessed explicitly in Figure 2, containing the posterior mean estimates (${\hat{\phi }}_{1},{\hat{\phi }}_{2}$) for each of the fixed combinations. The figure includes point estimates both using the A variant (left‐hand side) and P variant (right‐hand side) of the different methods. Here, the results refer to $\sigma_{\epsilon }^{2}=0.08$ (upper panels) and $\sigma_{\epsilon }^{2}=0.32$ (lower panels). The corresponding results using the other variance levels are given in the supplementary material (Figures S1–S9). For the two lowest levels of process variance, the estimation bias using CR‐INLA was slightly lower than using the other methods for all combinations of (*ϕ*_1_, *ϕ*_2_). When the process variance was increased, the different methods gave approximately the same point estimates. The bias was slightly larger using the P variants compared with the A variants. This was in correspondence with the higher averages of the RMSE values for the P variants, as already observed.

To further study coverage and RMSE for each of the 15 combinations, we computed a joint coverage being the proportion of times both of the estimated 95% credible intervals contained *ϕ*_1_ and *ϕ*_2_, respectively. We also computed a joint RMSE for both parameters, defined by

$$\text{RMSE}\left({\hat{\phi }}_{1},{\hat{\phi }}_{2}\right)=\sqrt{\frac{1}{M}\sum_{i=1}^{M}\sum_{k=1}^{2}{\left({\hat{\phi }}_{k}‐\phi_{k}\right)}^{2}}.$$

**FIGURE 3:**
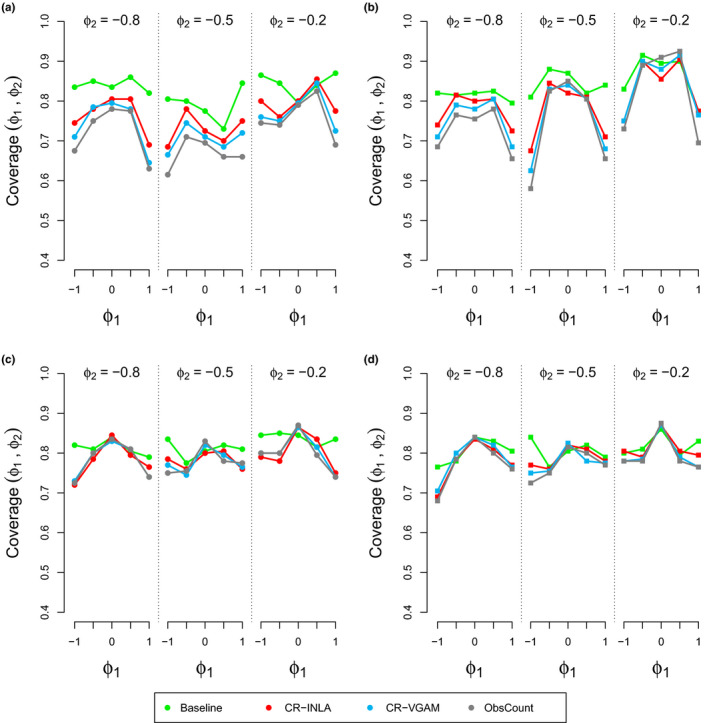
Joint coverage for different combinations of (*ϕ*_1_, *ϕ*_2_) for $\sigma_{\epsilon }^{2}=0.08$ (panels a and b) and $\sigma_{\epsilon }^{2}=0.32$ (panels c and d). A variants are represented on the left (panels a and c) and P variants on the right (panels b and d). The results were split into 3 sets (*ϕ*_2_ ∈ (−0.8, −0.5, −0.2), where each set includes the coverage results for *ϕ*_1_ ∈ (−1, −0.5, 0, 0.5, 1)

**FIGURE 4:**
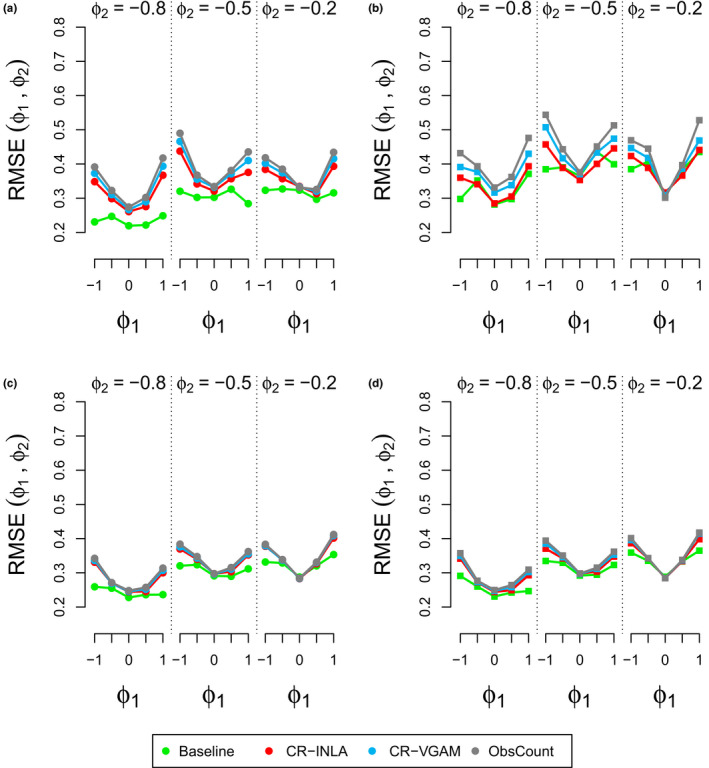
Joint RMSE for different combinations of (*ϕ*_1_, *ϕ*_2_) for $\sigma_{\epsilon }^{2}=0.08$ (panels a and b) and $\sigma_{\epsilon }^{2}=0.32$ (panels c and d). A variants are represented on the left (panels a and c) and P variants on the right (panels b and d). The results were split into 3 sets (*ϕ*_2_ ∈ (−0.8, −0.5, −0.2), where each set includes the RMSE results for *ϕ*_1_ ∈ (−1, −0.5, 0, 0.5, 1)

The results for coverage and RMSE are shown in Figures 3 and 4, respectively. Our results showed that the coverage was smallest and RMSE is largest when |*ϕ*_1_| = 1 in most combinations of the AR coefficients. CR‐INLA was seen to give the highest coverage and lowest RMSE for most of the combinations when $\sigma_{\epsilon }^{2}=0.08$, at least for the A variants. When $\sigma_{\epsilon }^{2}=0.32$, the results were very similar for all methods.

In summary, we can conclude that including capture history information improved the estimation of density dependence in process models having low process variance. Out of the tested methods, our suggested approach CR‐INLA performed best, followed by CR‐VGAM. For the given simulated data, the additional step of estimating log rates of a Poisson process resulted in larger RMSE.

Finally, we notice that both of the two AR coefficients were underestimated, and this bias increased with the absolute values of the coefficients.

The given simulation study was based on certain choices to illustrate a capture–recapture scenario using an AR(2) process model. Here, we have assumed independent capture probabilities for the two capture sessions. The given approach could have easily been adapted to other models given by Otis et al. (1978), such as to also include a behavioral effect. Longer time series would have improved the estimation results using all of the suggested methods, albeit being less realistic from an ecological point of view.

## 4 ESTIMATING DENSITY DEPENDENCE USING A REAL DATA SET

In this section, we estimated density dependence for a real capture–recapture data set of small mammals, collected at 20 different spatial locations over a period of 18 years. Our main focus was to assess density‐dependence estimates, studying how inclusion of capture history influenced the estimation. Using the CR‐INLA approach, we estimated capture probabilities by the regression model in (6), including individual‐specific covariate information and random effects. We proceeded to estimate the true abundances at each time point for each spatial location using (2). Finally, we fitted the AR(2) model to estimate density dependence and compared the results with using the methods CR‐VGAM and ObsCount. For all three methods, we assessed both the A and P variants.

### 4.1 Data description

**FIGURE 5:**
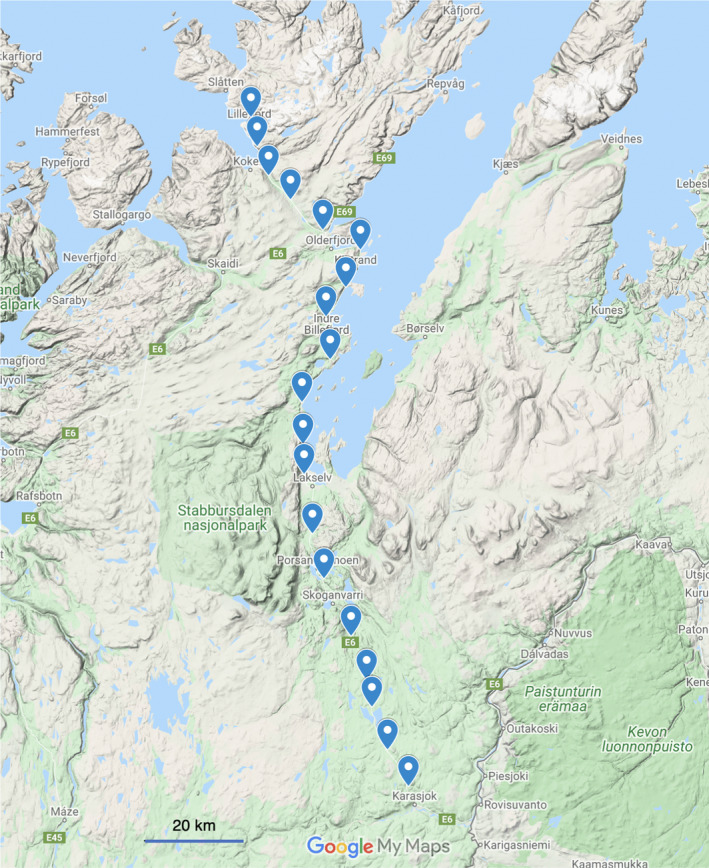
Stations distribution in Northern Norway, from Lillefjord to Karasjok. The station numbering goes along the north/south gradient, with station 1 being near Lillefjord and station 20 near Karasjok. This map was obtained from Google Maps

The data included a total of 3,090 gray‐sided voles, captured alive in the Porsanger region (latitude 70°N), in Northern Norway. The data were collected at 20 different stations, spaced evenly along 155 km of road (see Figure 5), in the period 2000–2017. Sampling was conducted twice a year, in spring and fall, and each capture session consisted of two visits. Two individual‐specific variables were recorded, including *weight* and *sex*. Animals captured dead during the first trapping session were excluded from the analysis.

### 4.2 Observation model selection, estimating capture probabilities

To estimate individual capture probabilities, we used the whole data set across time points and stations. Our first step was to select a reasonable observation model. Fitting the regression model in (6), we considered inclusion of the following variables

1. *Weight* (continuous standardized variable);
2. *Sex* (categorical variable for male or female);
3. *Season* (categorical variable for spring or fall);
4. *Station* (index variable for the evenly spaced stations);
5. *Time* (index variable for year)

To select which variables should be included, we evaluated different models using various information criteria. When applying CR‐INLA, we used the estimates for the deviance information criterion (DIC) (Spiegelhalter, Best, Carlin, & van der Linde, 2002) and Watanabe–Akaike's information criterion (WAIC) (Watanabe, 2010). When using CR‐VGAM, we used the estimates of Akaike's information criterion (AIC) (Akaike, 1973) and the Bayesian information criterion (BIC) (Schwarz, 1978).

An overview of the different models and the estimated information criteria is shown in Table 2, comparing the two methods for a total of 8 different models. The VGAM package does not allow for inclusion of random effect terms (Yee et al., 2015), which implies that *Time* could not be included in the CR‐VGAM model explicitly. Using INLA, we can straightforwardly include nonlinear effects of covariates. Applying the method CR‐INLA, we chose to model *Time* as a first‐order random walk process (rw1) (Rue & Held, 2005; Sørbye & Rue, 2014). Also, we considered to include *season* as a categorical covariate, both using CR‐INLA and CR‐VGAM. However, using the CR‐INLA approach, *season* is not included simultaneously with *time* to avoid confounding.

**TABLE 2:** Observation model selection for CR‐INLA and CR‐VGAM, using the selected information criteria. All values are given in comparison with the intercept model (1) for easier visualization. The lowest scores represent the best models, compromising goodness of fit with model complexity

		CR‐INLA		CR‐VGAM	
Model	Covariates	*DIC*	*WAIC*	*AIC*	*BIC*
1	Intercept	19,400	19,613	6,568	6,580
2	Weight	−149	−158	−8	−2
3	Weight + sex	−199	−217	−12	0
4	Weight + sex + season	−223	−242	−11	+8
5	Weight + sex + station	−213	−232	−10	+7
6	Weight + sex + time	−254	−278	–	–
7	Weight + sex + season + station	−249	−269	−9	+15
8	Weight + sex + station + time	−275	−300	–	–

The resulting optimal observation model for CR‐INLA, minimizing both DIC and WAIC, included all variables except *season*. The linear predictor as defined by (6) is here given by

$$\ln\left(E\left(Y_{\mathrm{ik}}\right)\right)=\ln\left(\mu_{\mathrm{ik}}\right)=\gamma_{k1}{\text{weight}}_{i}+\gamma_{k2}{\text{sex}}_{i}+\gamma_{k3}{\text{station}}_{i}+f\left({\text{time}}_{i}\right)+\beta_{i}+\epsilon_{i},\;i=1,\ldots ,n,\;k=1,\ldots ,3,$$

where *f*(time*_i_*) denotes the rw1 model, specifying a nonlinear random effect of time. In selecting an observation model for the CR‐VGAM approach, we observed rather small differences in the values of the goodness‐of‐fit criteria for the different models. The optimal observation model according to AIC included *weight* and *sex*, while BIC was minimized when only *weight* was included. In the case of vole species, *sex* is known to have an effect on detectability (Bryja et al., 2005), so we chose to include both *weight* and *sex* in estimating the capture probabilities.

**FIGURE 6:**
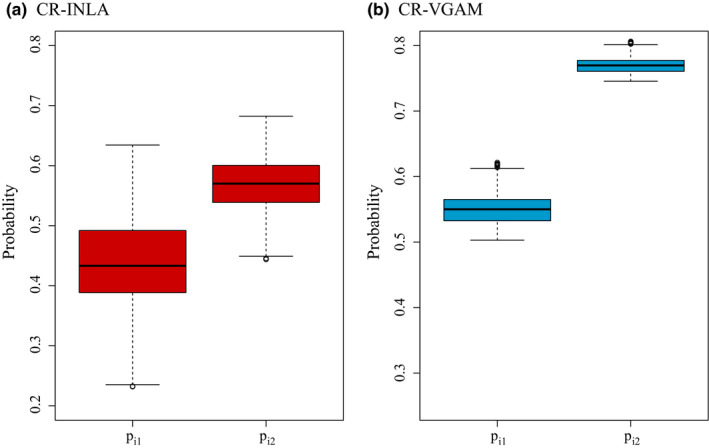
Estimates for p_1_ and p_2_ for the CR‐INLA (panel a) and CR‐VGAM (panel b) models

Figure 6 illustrates the distributions of the estimated capture probabilities for the two capture sessions, ${\left\{{\hat{p}}_{i1}\right\}}_{i=1}^{n}$ and ${\left\{{\hat{p}}_{i2}\right\}}_{i=1}^{n}$, using both CR‐INLA and CR‐VGAM. The mean capture probability is seen to increase on the second day using both methods. CR‐VGAM gave higher estimates of the capture probabilities, having a low variance for both days. Using CR‐INLA, the estimated individual capture probabilities showed more heterogeneity, having a larger variance for both days. Using the given estimated capture probabilities for the observed categories, we can estimate the probability that an individual is never captured, corresponding to category *c_i_*_0_ in (1). The resulting 95% percentile interval for *c_i_*_0_ was (0.19–0.32) using CR‐INLA. Using CR‐VGAM, the corresponding interval was (0.08–0.12).

### 4.3 Fitting the AR(2) process model to estimate density dependence

Given the estimates of the capture probabilities for each individual, we used the Horvitz–Thompson estimator to compute abundance at each time point for each station. We then fitted the AR(2) model to the resulting estimated log abundance, providing estimates of both direct and delayed density dependence. We split the time series into spring and fall, to account for a possible seasonal influence in the parameter estimation. This resulted in two time series of length *T* = 18 for each of the 20 stations. The AR(2) model was fitted using the three presented methods (CR‐INLA, CR‐VGAM, and ObsCount) using both the A and P variants. Station 9 did not have enough observations for the parameters to be estimated and was thus not included in the results.

**FIGURE 7:**
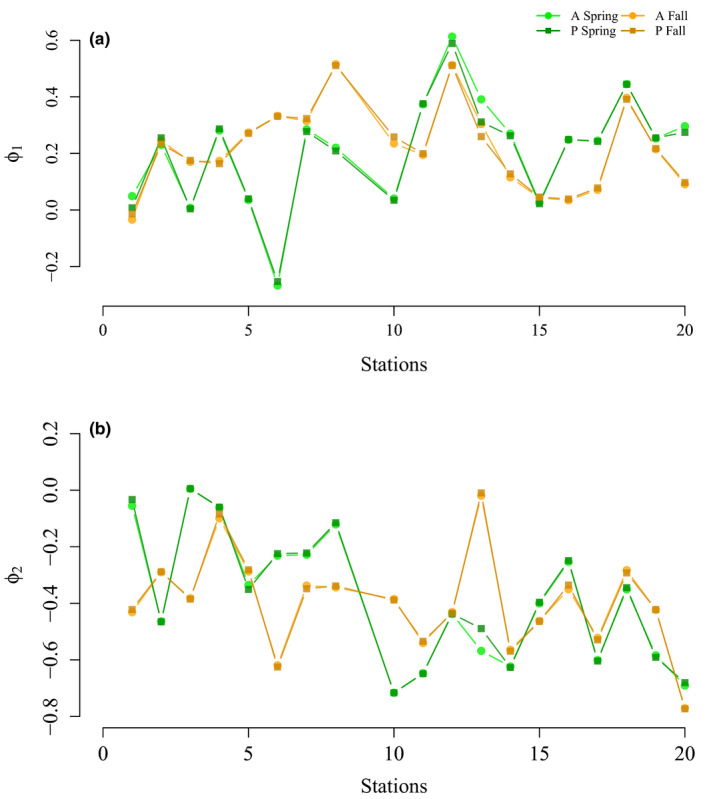
Estimates for *ϕ*_1_ (panel a) and *ϕ*_2_ (panel b) for the mean coefficients of both A and P variants, for the spring (green hue) and fall (orange hue) seasons separately

The main results are displayed in Figure 7, showing the posterior mean estimates of the AR coefficients for the two seasons, for variants A and P. The estimates of both direct and delayed density dependence were very similar using all the given methods and were thus lumped together (see Figures S11 and S12 for detailed values). Interestingly, the differences seen in the capture probability estimates between CR‐INLA and CR‐VGAM do not seem to have influenced the density‐dependence estimates. This is in correspondence with the simulation study in Section 3, as the process variance $\sigma_{\epsilon }^{2}$ for all of the stations was quite high, with the overall estimated average being ${\hat{\sigma }}_{\epsilon }^{2}=0.9$. This value likely corresponds to an overestimation of $\sigma_{\epsilon }^{2}$, with the true average being likely significantly lower and closer to 0.6 (see Figure S10 for bias estimates at different variance levels). In both spring and fall, the estimates of *ϕ*_1_ varied from around −0.25 to 0.6, whereas the estimates of *ϕ*_2_ ranged from around 0 to −0.8. For all stations, except 3 and 13, the estimated time series showed a semi‐periodic behavior. We also notice that the AR(2) coefficients varied with season for the same station, which suggests a seasonal effect in the density dependence. Additionally, during both seasons, the results indicate a decreasing trend in the value of *ϕ*_2_ along the given transect (from coast to inland).

## 5 DISCUSSION

The main goal of this paper was to assess the importance of including capture heterogeneity in the estimation of density dependence, thus incorporating sampling error in the observation model. To investigate this, we performed an extensive simulation study in which we generated AR(2) time series, representing the true log abundance of an animal population, and simulated a CR sampling scenario from that population. We then tested the performance of different methods, both including capture history information and disregarding it. For the first method, CR‐INLA, we defined an observation model to estimate individual capture probabilities through a multinomial likelihood and followed it with a Horvitz–Thompson estimate of the true abundance. The second method, CR‐VGAM, used the existing VGAM methodology to estimate abundance from CR data, establishing it as a control method. Finally, we compared these two methods with a simple (yet common) approach, disregarding the capture history information (effectively assuming a homogeneous capture process), to estimate the true autoregressive coefficients from the observed counts directly. We further investigated the assumption of using a Poisson distribution for the capture data, fitting the AR(2) process to the estimated log rates. This was chosen as an example of an observation model used in the ubiquitous state‐space models, where the observation model typically assumes some type of homogeneous capture process, such as Poisson or log normal.

Based on our simulation study, we found that incorporating capture history information was important in estimating density dependence for AR(2) models with process variance below 0.16. In such scenarios, both methods including capture history outperformed the method disregarding it, with reduced estimation bias and improved parameter coverage (8% higher in CR‐INLA (A) compared with ObsCount (A) for the lowest tested process variance; see Table S1). However, in scenarios with a large process variance, the methods that estimated capture probability did not stand out, producing extremely similar results compared with the observed counts approach. Furthermore, parameter estimates for both AR coefficients were generally biased toward 0, using all the methods, increasingly underestimating the absolute values of the parameters. In the context of quasi‐periodic dynamics described by an AR(2) process, this means underestimating the strength of direct *ϕ*_1_ and delayed *ϕ*_2_ density dependence, and overestimating the process variance of the AR(2) model (see Figure S10).

The data collected in Porsanger showcased vole populations with very large fluctuations in abundance, as is typical of such systems (Cornulier et al., 2013; Henttonen & Hanski, 2000). Moreover, the estimated capture probabilities were relatively high, resulting in a relatively small bias when comparing the observed counts and the estimated abundance. This resulted in all methods, and respective variants, producing similar results—this could have been expected given the observation variance is, in that case, only a minor component of the total variance. Other populations, such as large mammals, may show much smaller abundance fluctuations and therefore a larger contribution of the observation error to the total variance (e.g., Besbeas & Morgan, 2019). Moreover, in the case of other animal groups, such as snakes, capture probabilities are often very low (Rose, Wylie, Casazza, & Halstead, 2018), and could potentially lead to a larger bias corrections in density‐dependence estimates by accounting for capture heterogeneity (Fletcher et al., 2011), although we do not explicitly test this in this paper.

Extending our approach to other observation process models (e.g., spatial capture–recapture models (Royle, Fuller, & Sutherland, 2017), including individual heterogeneity (Efford & Mowat, 2014), would provide a general approach to reducing biases in population dynamic models. One disadvantage of the CR‐INLA method is that it would be cumbersome to apply in CR data sets with more than 3 days, given the data expansion necessary to fit multinomial likelihoods in INLA, where all the category combinations, observed and not, must be present. This could potentially be automatized as in Bayesian fitting of capture–mark–recapture models (McCrea, 2014).

Two limitations of our simulation study were pointed out during the revision process of this manuscript. Given the complex nature of the simulation setup, involving 4 methods in 2 variants, and 75 combinations of parameters, the running time proved to be lengthy. Even when running in parallel, we needed roughly 150 hr to obtain the full results, from running 200 simulations per unique combination of parameters. Time thus became a constraint and prevented us from running a higher number of simulations, such as 500 or even 1,000. Nonetheless, we believe our results show true patterns as we noticed early convergence in both coverage and RMSE, from around 50 simulations. Moreover, because we decided to split the results by process variance level, our aggregated averages, displayed in Table 1, combine 3,000 simulations for each method. In addition, it was noted that we did not propagate the uncertainty from our first modeling step into the second. We recognize that this would be a definite advantage, working with a Bayesian framework, and we have now investigated this. We first generated 200 posterior samples from the fitted multinomial model, and then, we fitted the AR(2) model to these samples. The resulting variance in estimating the AR coefficients was very small. This can be seen with an example displayed in Figure S13. The upper panels (a and b) show the posterior sampling distributions for the differences in the specific coefficients related to the categories of the multinomial model. These are used to estimate the abundances at each time point (panel c). The resulting distributions for the AR coefficients (panel d and e) are very narrow giving standard deviations smaller than 0.01. This is much smaller than the standard deviation in estimating the coefficients of independently generated AR(2) time series. Therefore, we realized we would not gain much from the uncertainty propagation, also taking into consideration the large amount of additional running time required.

In summary, we have found that capture–recapture information given by two capture events contributes to improve density‐dependence estimates of AR(2) models with low process variance. In such cases, we recommended that capture heterogeneity is accounted for in the observation model, as it can constitute an important part of the total sampling error. Further analyses are required to assess how more capture events could impact process estimation.

## CONFLICT OF INTEREST

None declared.

## AUTHOR CONTRIBUTION

**Pedro Guilherme Nicolau:** Conceptualization (equal); Data curation (lead); Formal analysis (equal); Investigation (equal); Methodology (equal); Resources (supporting); Software (supporting); Validation (equal); Visualization (equal); Writing‐original draft (lead); Writing‐review & editing (equal). **Sigrunn Sørbye:** Conceptualization (equal); Formal analysis (equal); Investigation (equal); Methodology (equal); Project administration (equal); Software (equal); Supervision (lead); Validation (equal); Writing‐original draft (equal); Writing‐review & editing (equal). **Nigel Yoccoz:** Conceptualization (lead); Funding acquisition (lead); Investigation (equal); Methodology (supporting); Project administration (equal); Resources (lead); Supervision (supporting); Writing‐original draft (equal); Writing‐review & editing (equal).

### OPEN RESEARCH BADGES

This article has been awarded Open Materials, Open Data Badges. All materials and data are publicly accessible via the Open Science Framework at https://doi.org/10.5061/dryad.fj6q573rr.

## Supporting information

Supplementary Material

## Data Availability

The simulation code used to obtain the results in this paper and the real data set used to test the methods have been submitted to Dryad (https://doi.org/10.5061/dryad.fj6q573rr).
