# Supplementary material for: Incorporating capture heterogeneity in the estimation of autoregressive coefficients of animal population dynamics using capture–recapture data
Source: Ecol Evol. 2020 Aug 31;10(23):12710–26. doi: 10.1002/ece3.6642 (PMC7713978; doi:10.1002/ece3.6642)
Supplement: Supplementary file 1 — Supplementary Material [file ECE3-10-12710-s001.docx]

# Appendix A - Simulation results


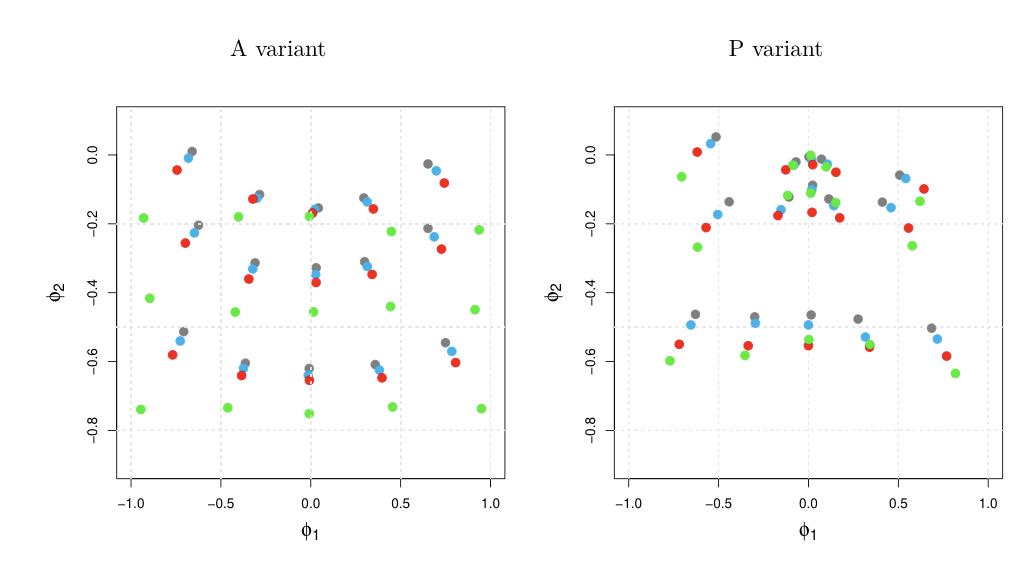


Figure A1 Estimates of the different methods for σ^2^= 0.04


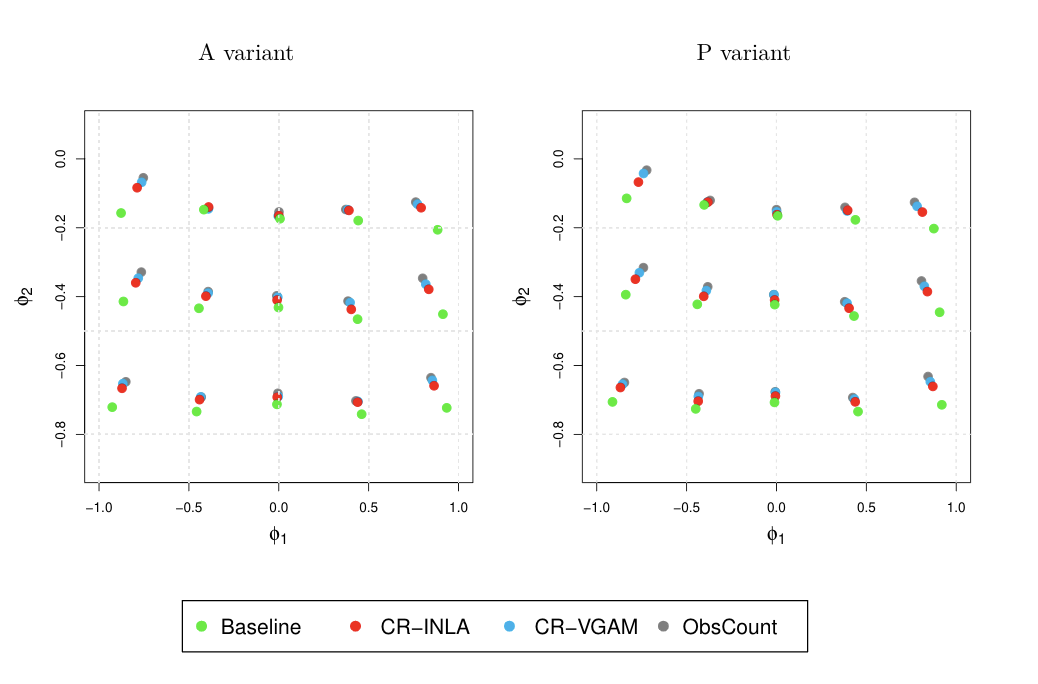


Figure A2 Estimates of the different methods for σ^2^= 0.16


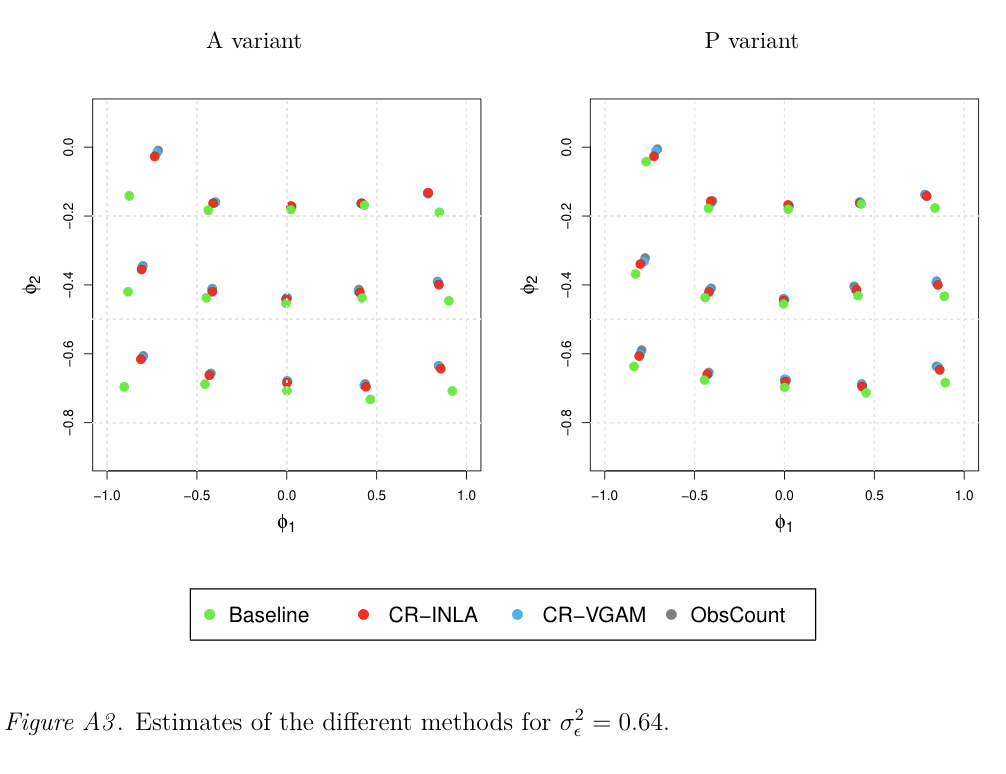


Figure A3. Estimates of the different methods for $\sigma_{\epsilon}^{2}=0.64$.


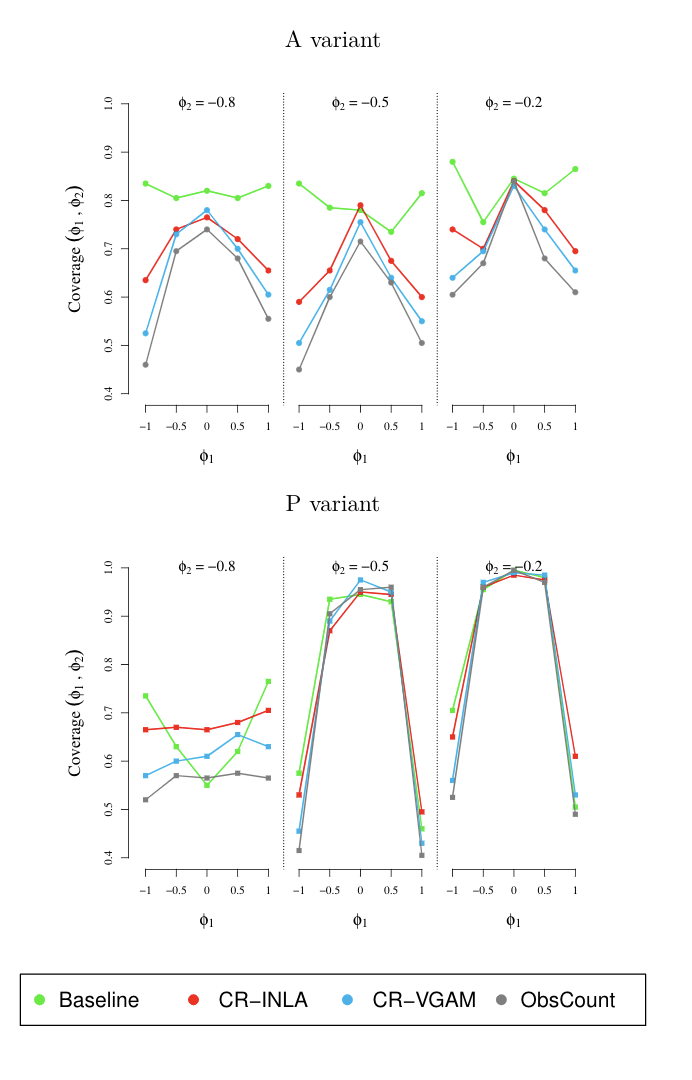


Figure A4. Coverage for different combinations of ($\phi_{1}$,$\phi_{2}$) for $\sigma_{\epsilon}^{2}=0.04$ in both variants.


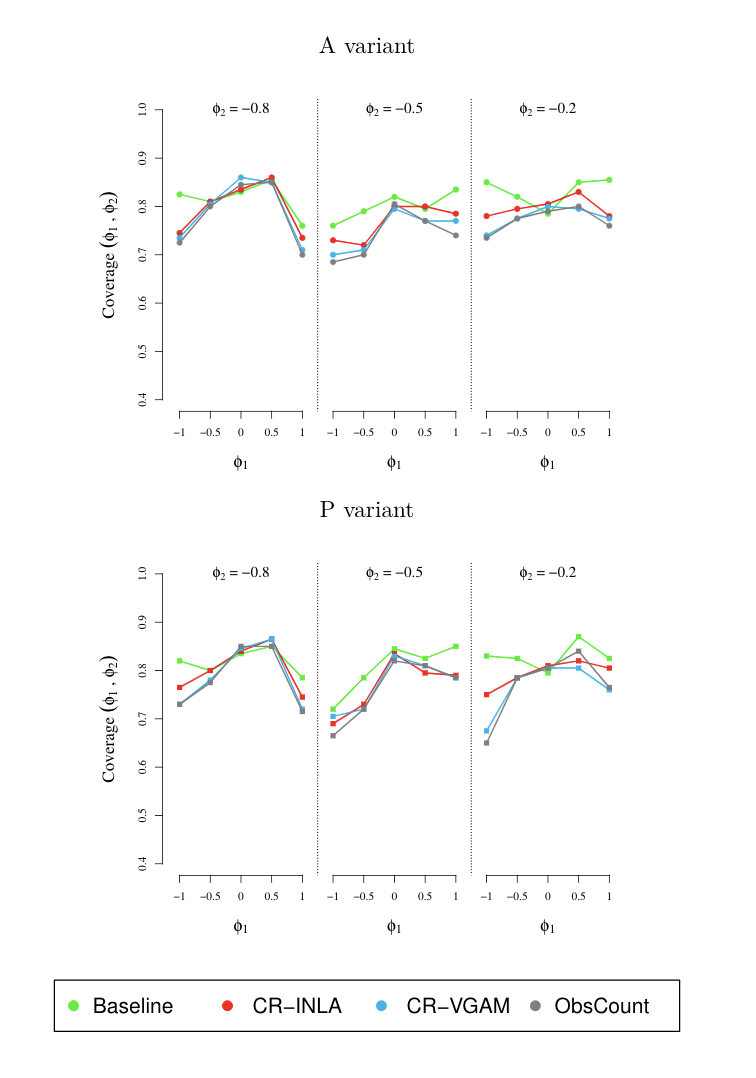


Figure A5. Coverage for different combinations of ($\phi_{1}$,$\phi_{2}$) for $\sigma_{\epsilon}^{2}=0.16$ in both variants.


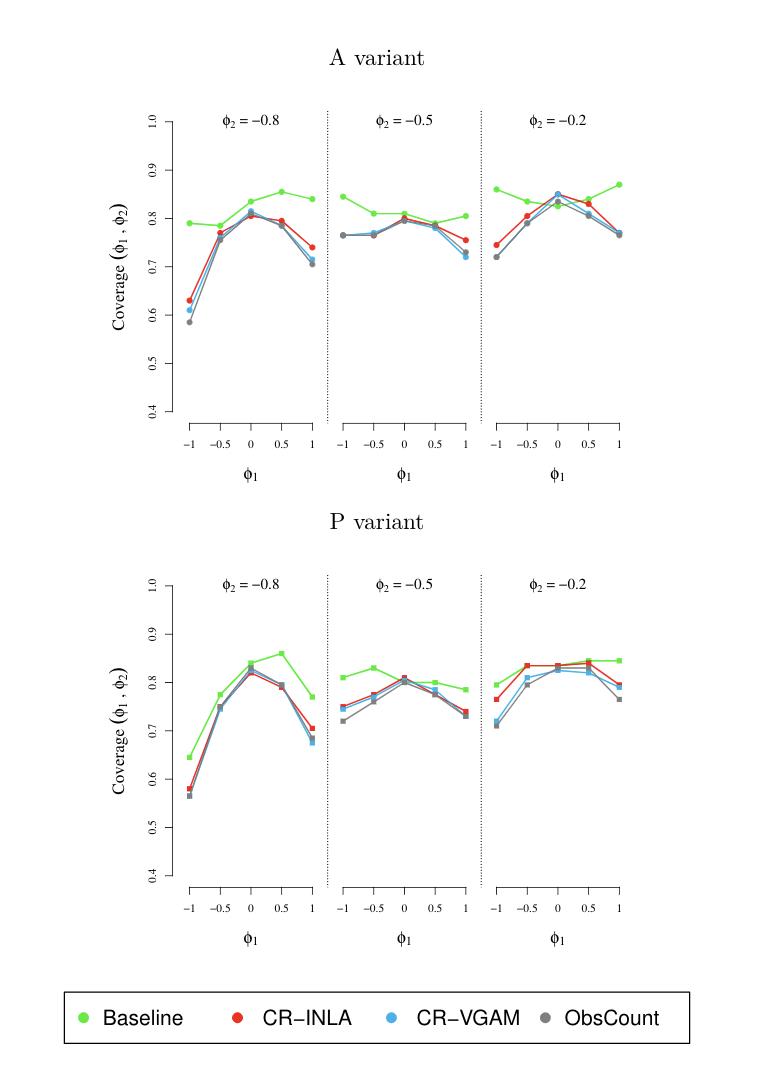


Figure A6. Coverage for different combinations of ($\phi_{1}$,$\phi_{2}$) for $\sigma_{\epsilon}^{2}=0.64$ in both variants.


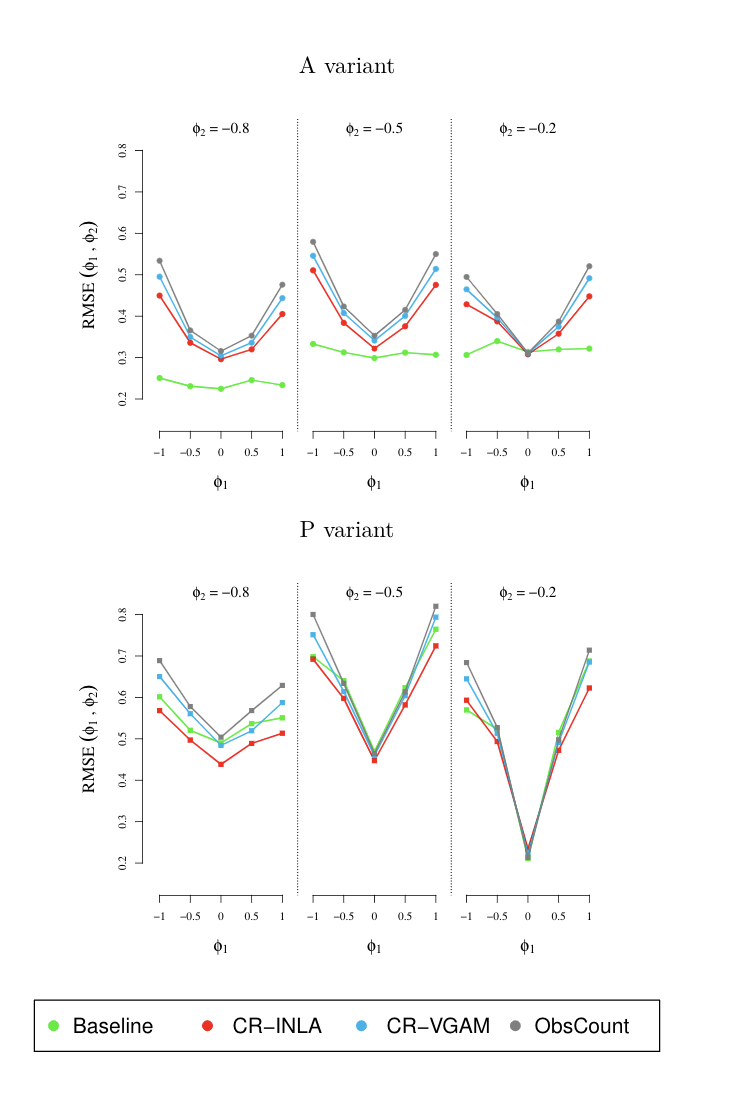


Figure A7. RMSE for different combinations of ($\phi_{1}$,$\phi_{2}$) for $\sigma_{\epsilon}^{2}=0.04$ in both variants.


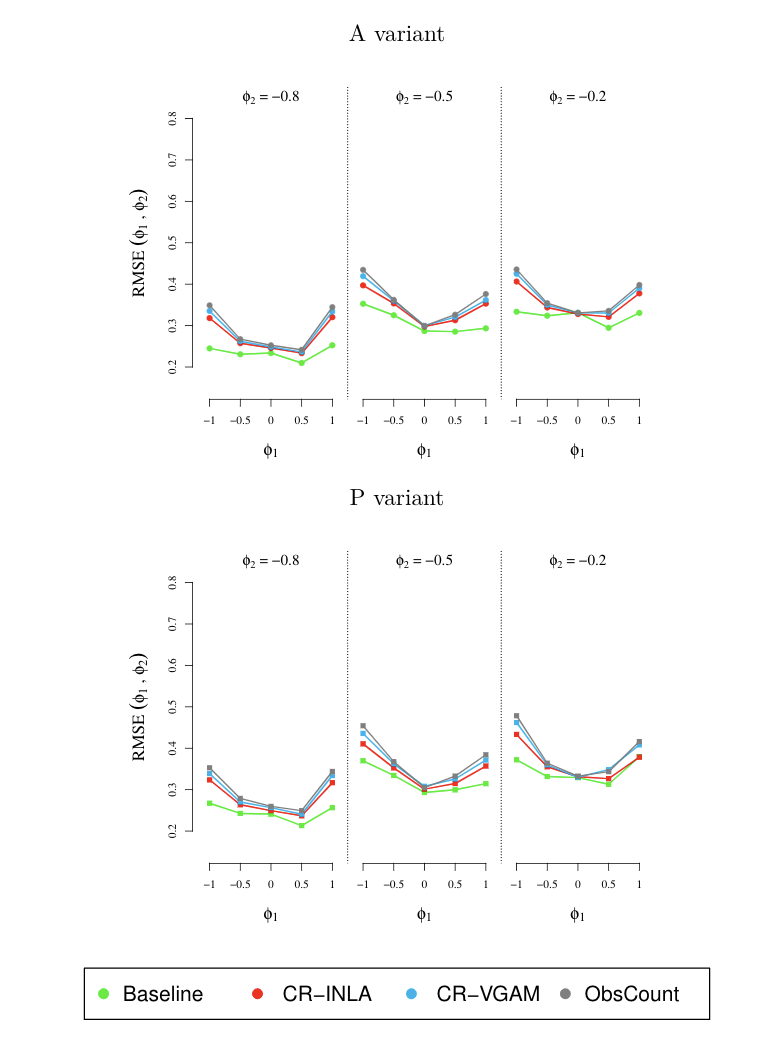


Figure A8. RMSE for different combinations of ($\phi_{1}$,$\phi_{2}$) for $\sigma_{\epsilon}^{2}=0.16$ in both variants.


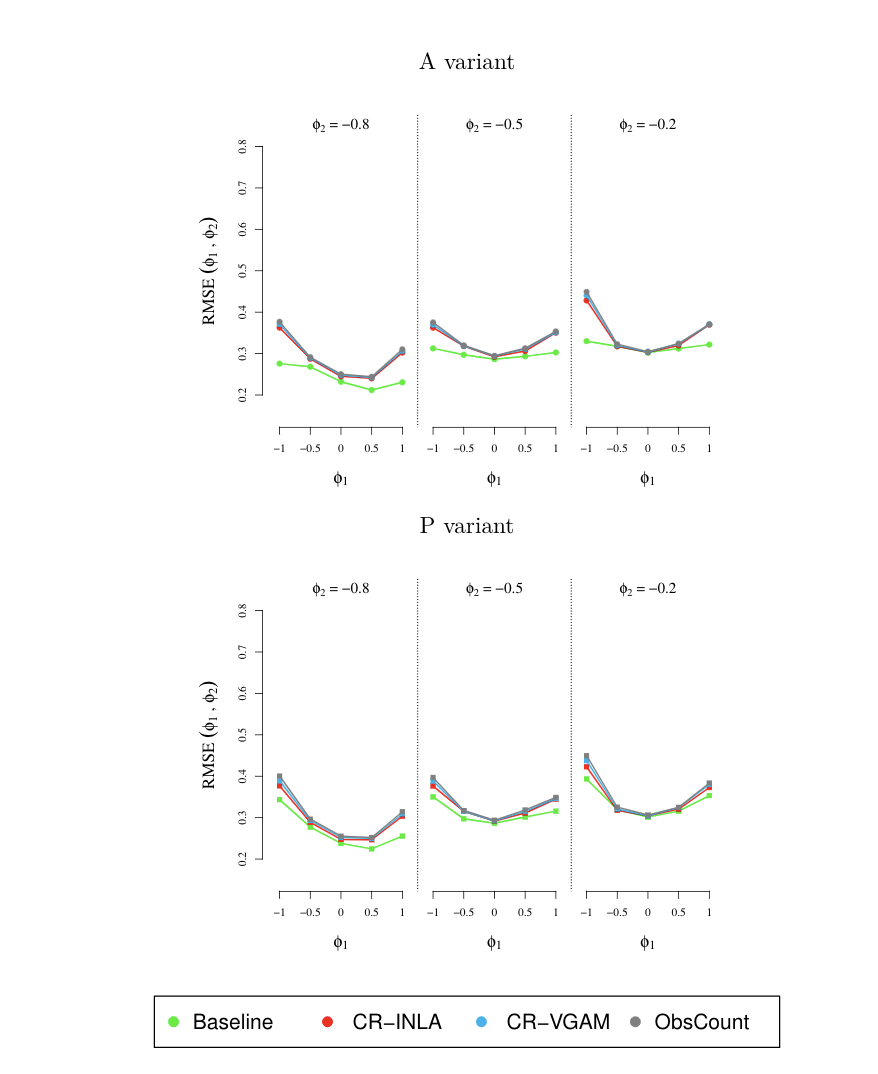


Figure A9. RMSE for different combinations of ($\phi_{1}$,$\phi_{2}$) for $\sigma_{\epsilon}^{2}=0.64$ in both variants.

Figure A10: Mean process variance estimate for the A variants in the simulation exercise, across all combinations of parameters. The dashed grey line corresponds to the theoretical optimum. The green line provides the practical optimum. The three methods provide similar estimates of the process variance, increasingly overestimating it for larger $\sigma_{\epsilon}^{2}$ values.

Table A1 Values of average joint coverage and joint RMSE for all combinations of simulations for the four different methods, in 5 levels of variance $\sigma_{\epsilon}^{2}$. (A) columns represent the log-Abundance variants, while (P) columns show values for the log-Poisson rate variants.

|  |  |  |  |  |  |
| --- | --- | --- | --- | --- | --- |
|  |  | *Joint Coverage* | | *Joint RMSE* | |
| **Method** | $\boldsymbol{\sigma}_{\boldsymbol{\epsilon}}^{\boldsymbol{2}}$ | ***A*** | ***P*** | ***A*** | ***P*** |
| Baseline | 0.04 | 0.81 | 0.75 | 0.41 | 0.75 |
| CR-INLA |  | 0.71 | 0.76 | 0.54 | 0.72 |
| CR-VGAM |  | 0.66 | 0.72 | 0.58 | 0.77 |
| ObsCount |  | 0.63 | 0.69 | 0.61 | 0.80 |
| Baseline | 0.08 | 0.82 | 0.84 | 0.40 | 0.51 |
| CR-INLA |  | 0.76 | 0.80 | 0.48 | 0.53 |
| CR-VGAM |  | 0.74 | 0.78 | 0.51 | 0.57 |
| ObsCount |  | 0.72 | 0.77 | 0.53 | 0.60 |
| Baseline | 0.16 | 0.82 | 0.82 | 0.41 | 0.43 |
| CR-INLA |  | 0.79 | 0.79 | 0.46 | 0.46 |
| CR-VGAM |  | 0.77 | 0.77 | 0.47 | 0.48 |
| ObsCount |  | 0.77 | 0.77 | 0.48 | 0.49 |
| Baseline | 0.32 | 0.82 | 0.81 | 0.41 | 0.43 |
| CR-INLA |  | 0.79 | 0.79 | 0.45 | 0.45 |
| CR-VGAM |  | 0.79 | 0.79 | 0.45 | 0.46 |
| ObsCount |  | 0.79 | 0.78 | 0.46 | 0.47 |
| Baseline | 0.64 | 0.83 | 0.80 | 0.40 | 0.43 |
| CR-INLA |  | 0.77 | 0.77 | 0.45 | 0.45 |
| CR-VGAM |  | 0.76 | 0.76 | 0.46 | 0.46 |
| ObsCount |  | 0.76 | 0.76 | 0.46 | 0.47 |

# Appendix B - Real data results


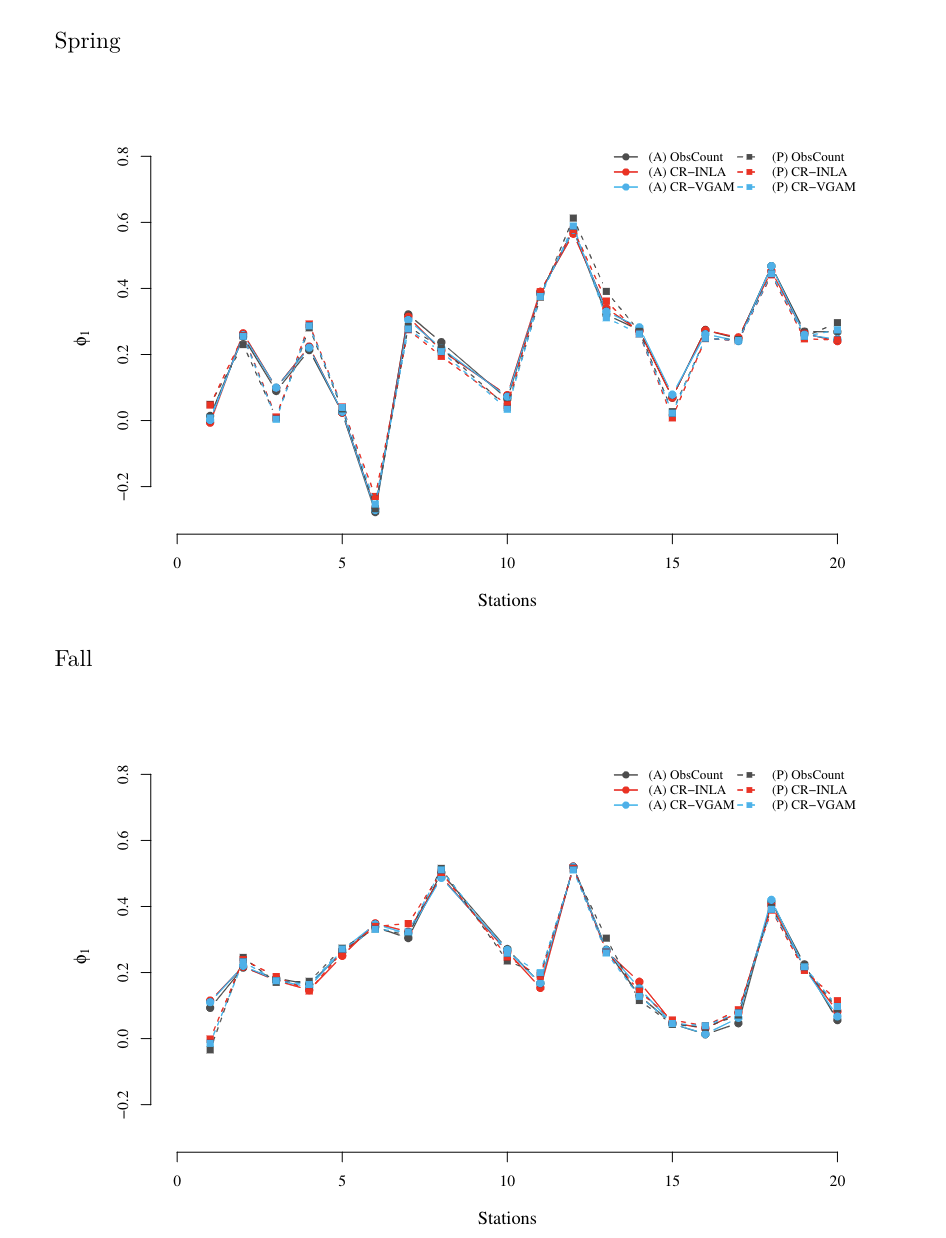


*Figure B1. Estimates for φ1 for the different methods in both variants, per season.*


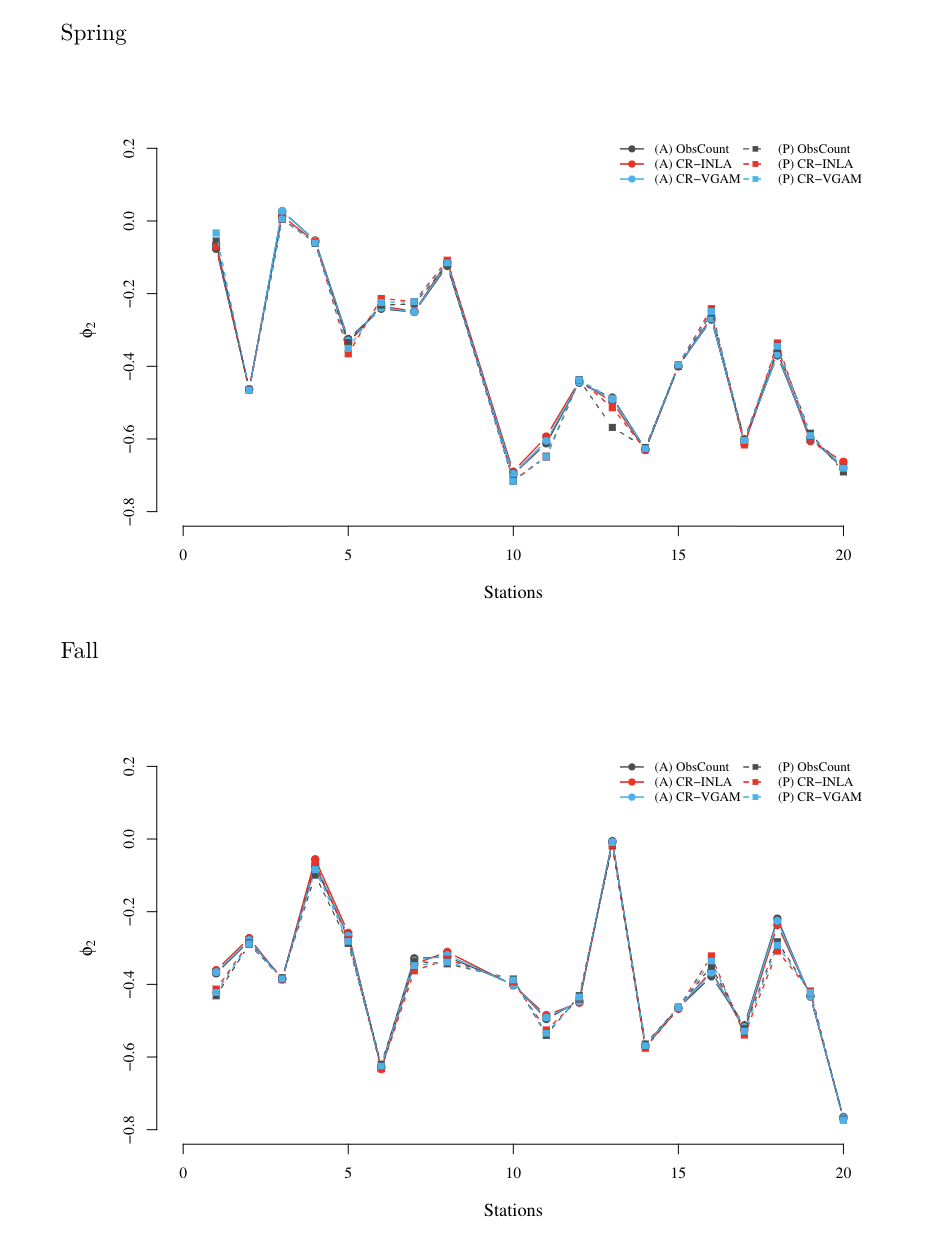


*Figure B2. Estimates for φ2 for the different methods in both variants, per season.*

# Appendix C - Propagating uncertainty example


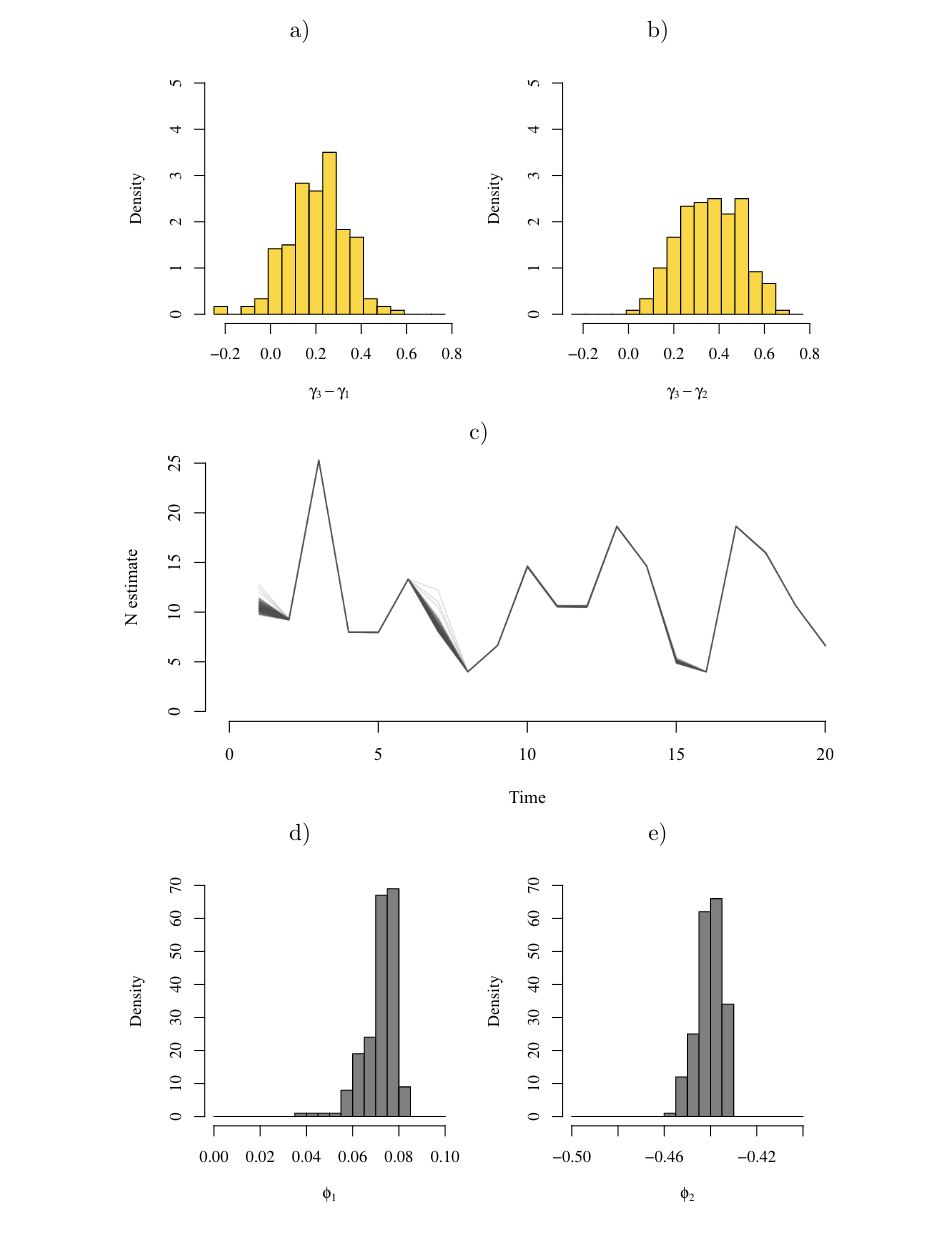


Figure C1: Example of propagating uncertainty from the estimation of the $\gamma$-coefficients of the multinomial model (a and b) to the estimation of $\phi_{1}$ and $\phi_{2}$ with CR-INLA for one simulation setting. These plots show an example of propagating uncertainty from the estimation of the $\gamma$-coefficients, used to estimate the capture probabilities in  [(9)](#eq:capture-prob1) and [(10)](#eq:capture-prob2), to estimating abundance and the AR coefficients. The posterior Monte Carlo samples for the $\gamma$-coefficients were obtained using the function inla.posterior.sample for the fitted multinomial model. The figure illustrates error propagation for a single simulated dataset with true parameters $\{\phi_{1}=0.2,\phi_{2}=-0.5,\sigma_{\epsilon}^{2}=0.08\}$, using 200 Monte Carlo samples. Panels a) and b) show the distribution of the differences between the $\gamma$-coefficients. Panel c) shows the resulting overlapped estimates for the abundance $\overset{̂}{N}$, while panels d) and e) give the resulting distributions for $\phi_{1}$ and $\phi_{2}$. These distributions are very narrow, giving standard deviations equal to 0.007 and 0.005 for $\phi_{1}$ and $\phi_{2}$, respectively. This is a consequence of the very low variation in the estimated abundance in panel c).
